# Osteogenic Differentiation Induced by Dental Pulp Stem Cells Secretome: A Dose‐Dependent Comparative Study

**DOI:** 10.1002/cre2.70395

**Published:** 2026-06-23

**Authors:** Mahdi Kadkhodazadeh, Reza Amid, Aida Kheiri, Sayna Shamszadeh, Forough Shams

**Affiliations:** ^1^ Department of Periodontics, School of Dentistry Shahid Beheshti University of Medical Sciences Tehran Iran; ^2^ Dental Research Center, Research Institute of Dental Sciences Shahid Beheshti University of Medical Sciences Tehran Iran; ^3^ Iranian Center for Endodontic Research, Research, Institute of Dental Sciences Shahid Beheshti University of Medical Sciences Tehran Iran; ^4^ Department of Medical Biotechnology, School of Advanced Technologies in Medicine Shahid Beheshti University of Medical Sciences Tehran Iran

**Keywords:** mesenchymal stem cells, periodontal ligament, regeneration, secretome, tissue engineering

## Abstract

**Objectives:**

To investigate the effects of dental pulp stem cell‐derived conditioned medium (DPSC‐CM) on the proliferation, migration, and osteogenic differentiation of periodontal ligament stem cells (PDLSCs). Primary goals were to (i) identify the optimal DPSC‐CM' concentration and (ii) evaluate its effect both independently (under Growth Medium, [GM]) and synergistically (under Osteogenic Medium [OM]).

**Materials and Methods:**

DPSC‐CM was collected, and an initial screen was conducted to determine their maximum non‐toxic concentration (MNTS). For functional assays, three non‐cytotoxic concentrations (V/V) (10% [CM‐10], 30% [CM‐30], 50% [CM‐50]) were selected. While cytokine release profile, cell proliferation, and migration were assessed under GM conditions, differentiation was evaluated over 14d under both GM and OM conditions (*p* < 0.05).

**Results:**

The MNTS was established as CM‐50. DPSC‐CM promoted all cellular activities in a non‐linear, concentration‐specific manner. CM‐10 demonstrated the most beneficial effects; it yielded the highest levels of TGF‐β1 and VEGF secretion (*p* < 0.05), and significantly increased proliferation, alkaline phosphatase activity, and expression of osteogenic/angiogenic markers than CM‐50 and CM‐30 over time. However, peak cell migration was observed on Day 2 for both CM‐30 and CM‐10. While DPSC‐CM alone enhanced osteogenesis, the highest induction was achieved under OM conditions.

**Conclusions:**

DPSC‐CM is a potent osteoinductive agent, enhancing proliferation, migration, and differentiation in a non‐linear concentration‐specific manner. The lowest concentration proved optimal for their proliferative and osteogenic properties, suggesting a Hormesis‐like effect where higher doses may become sub‐optimal due to inhibitory factors.

## Summary

- The secretome of dental pulp stem cells is a potent osteoinductive agent.
- It enhanced migration/osteoblast‐differentiation of periodontal‐ligament stem cells.
- The optimal dose is 10% with a hormesis‐like effect.
- The higher doses> 30% might have inhibitory factors.

## 1 Introduction

Periodontitis is a chronic inflammatory condition that leads to the destruction of soft tissues (e.g., gingiva or periodontal ligament) and hard tissues (e.g., marginal alveolar bone), ultimately resulting in tooth loss. Since periodontal inflammation can lead to irreversible destruction of tooth‐supporting tissues, several treatment modalities have been proposed with the aim of enhancing complete regeneration of lost tissues and restoring their functions (Jagadale et al. 2025). The three main components of bone tissue engineering are stem cells, biomaterials, and signaling molecules (Jagadale et al. 2025). Allografts, xenografts, and synthetic bone graft substitutes serve as osteoconductive scaffolds that can improve cell adhesion and differentiation, with autogenous bone graft being the gold standard due to its osteogenic, osteoinductive, and osteoconductive properties (Amid et al. 2021). Since periodontal damage is mostly irreversible, several periodontal regeneration therapies have been proposed to arrest these periodontal lesions and prevent their progression, thereby aiming to fully regenerate lost tissue and restore its functions. Regeneration of bone defects remains a substantial clinical challenge. Current treatment strategies typically include the application of autografts, allografts, xenografts, and synthetic bone grafts. Although autologous bone grafts are considered the gold standard, their application requires harvesting bone from the patient, which can lead to donor site morbidity, limited supply, and the need for additional surgical interventions. Allografts can be effective for managing bone defects and fractures, but they carry risks such as immune rejection, disease transmission, and inadequate osteointegration (Lyons et al. 2020). These limitations underscore the necessity for alternative therapies that can more effectively enhance bone regeneration (Du et al. 2015).

Another important aspect of bone regeneration is the presence of stem cells. Over the past decades, stem cell‐based therapies, particularly those employing mesenchymal Stem Cells (MSCs), have shown promising results due to their specific characteristics, including ease of accessibility, multilineage differentiation capacity, and immunomodulatory effect (Shekatkar et al. 2024). Research has shown that transplanting MSCs into a periodontal defect can positively influence periodontal regeneration (Bright et al. 2015). Beyond periodontal regeneration, a recent systematic review showed that modification of implant surfaces through the application of MSCs enhanced osseointegration in vivo. This increase in bone‐to‐implant contact might be the result of different growth factors that induced osteoblastic differentiation and new bone formation (Sayed et al. 2021).

However, stem cell therapies have some drawbacks, such as poor survival rate, the risk of tumorigenicity, unpredictable surface molecule expression, the potential risk of pathogenic factors' transition, and high costs (Iwasaki et al. 2022).

Previous studies have advocated the role of MSCs‐derived secretome (conditioned medium [CM]) as a promising alternative therapeutic strategy. The secretome refers to the trophic factors, including cytokines, chemokines, growth factors, and extracellular vesicles (Teixeira et al. 2016; Wang et al. 2022). This therapeutic approach involves administering the secreted factors during paracrine action, avoiding the risks associated with stem‐cell therapies (Rajan et al. 2016). Moreover, it possesses immunomodulatory and regeneration potential (Chouaib et al. 2022; Lin et al. 2021). A recent study by Kumar et al. examined the contribution of proteins pertaining to osteogenic lineage in the dental stem cells. Their findings suggest that dental pulp stem cells (DPSCs) tend to have more osteogenic potential than bone marrow stem cells and dental follicle stem cells, attributed to the presence of proteins like Sterile alpha motif domain containing protein 9 and bone morphogenic protein 7. It seems that the potent tropic factors at the site of tissue injury can modulate the microenvironment and activate nearby resident stem cells in a paracrine manner, eliciting these progenitors to initiate repair/regeneration processes (Amghar‐Maach et al. 2019; Kumar et al. 2018).

Two studies have reported the osteogenic effect of DPSCs‐derived CM (DPSC‐CM) on the general precursor, such as pre‐osteoblast. Given its relevance to the repair/regeneration of bony defects in periodontitis, it is essential to investigate the osteoinductive effects of secretome derived from DPSCs on periodontal ligament stem cells (PDLSCs). Therefore, as a first step toward this direction, the present study aimed to address three crucial questions: (1) Can DPSC‐CM induce osteoblast differentiation of PDLSCs? (2) Does DPSC‐CM function independently of osteogenic media? (3) Are the osteoinductive effects dose‐dependent?

## 2 Materials and Methods

### 2.1 Ethical Approval

The current research was approved by the Ethics Committee of the Dental School at Shahid Beheshti University of Medical Sciences under approval number IR.SBMU.DRC.REC*.1402.079*. Informed consent was obtained from all patients involved in the study.

### 2.2 MSCs Isolation

DPSCs were obtained from the Research Institute of Dental Sciences, Tehran, Iran. PDLSCs were isolated from sound extracted premolars of patients aged 16–20 years at the Oral and Maxillofacial Surgery Department at Shahid Beheshti Dental School. The periodontal ligaments were carefully scraped from the middle third of the tooth roots, rinsed twice with phosphate‐buffered saline (PBS), and digested in a collagenase I solution (3 mg/mL) for 60 min at 37°C. The digested tissues were then seeded in a T‐25 tissue culture flask (Corning, USA). Cells that adhered to the flask were cultured in Dulbecco's Modified Eagle Medium (DMEM, Stem Cell Technology, Bio idea, Iran), supplemented with 10% fetal bovine serum (FBS, Gibco, Australia), and 1% penicillin‐streptomycin (Sigma‐Aldrich, St Louis, USA) at 37°C/5% CO_2_. Once the cells reached 80%–90% confluency, they were passaged. For the current experiments, primary cells at 3rd to 4th passage were used.

### 2.3 Stem Cell Characterization

PDLSCs at the 3rd passage were characterized for the presence of MSC markers (CD90 and CD105) and absence of negative hemopoietic markers (CD34 and CD45) using flow cytometry.

PDLSCs (2 × 10^5^ cells) were resuspended in PBS with 0.1% FBS, containing a 1:100 dilution of the following fluorescein isothiocyanate (FITC)‐conjugated antibodies: CD 90 (BD Biosciences, San Jose, CA, Cat# 740, 786, PRD: AB_2740449), CD 105 (BD Biosciences, San Jose, CA, Cat# 562, 380, PRD: AB_11154054), CD34 (BD Biosciences, San Jose, CA, Cat# 340,62, PRD: AB_400150), and CD‐45 (BD Biosciences, San Jose, CA, Cat# 610,265, PRD: AB_397660). The mixture was incubated for 1 h on dry ice in the dark. The cell suspension was then washed twice with PBS and analyzed. Data were analyzed using CellQuest software version 7.5.3 (BD Biosciences, San Jose, CA).

### 2.4 DPSC‐CM Collection

DPSCs at the 3rd passage were cultured in 75 cm^2^ flasks (5000–6000 cells/cm^2^). Upon reaching approximately 70%–80% confluency, the medium was replaced with a fresh culture medium (without serum). Culture flasks were kept at an incubator (37°C, 5% CO_2)_ for 48 h. Metabolic activity, as well as cell morphology, was monitored to ensure that the cells did not undergo serum starvation. To obtain secretome, the culture medium was collected, centrifuged at 3000 rpm for 5 min to remove cell debris and apoptotic bodies, and filtered through a 0.22‐µm filter. Subsequently, the filtered secretome was concentrated using a 5 kD ultrafiltration tube and centrifuged at 5000*g* at 4°C for 40 min. All secretomes were stored at −80°C.

### 2.5 Total Protein of DPSC‐CM

The total protein concentration of the crude DPSC‐CM (100% V/V) was determined using the bicinchoninic acid (BCA) Protein Assay kit (Thermo Fischer Scientific, Milan, Italy), according to the manufacturer's instructions. The protein concentration was extrapolated from a calibration curve of concentration vs. absorbance using standard Bovine Serum Albumin (BSA) solution, employing a third‐order polynomial equation (*R* ^2^ = 0.99).

### 2.6 Preparation of Different Concentrations of DPSC‐CM

The stock DPSC‐CM was diluted with GM to achieve different concentrations (expressed as % V/V) as follows: CM‐90 (ratio GM: CM [10:90]), CM‐80 (ratio GM: CM [20:80]), CM‐70 (ratio GM: CM [30:70]), CM‐60 (ratio GM: CM [40:60]), CM‐50 (ratio GM: CM [50:50]), CM‐40 (ratio GM: CM [60:40]), CM‐30 (ratio GM: CM [70:30]), CM‐20 (ratio GM: CM [80:20]), and CM‐10 (ratio GM: CM [90:10]).

### 2.7 Cell Viability/Proliferation Assay

The overall effect of DPSC‐CM on PDLSCs' metabolic activity and viability was assessed using the MTT assay. This section was divided into two parts: cytotoxicity screening and proliferation assay.

### 2.8 Cytotoxicity Screening

This screening aimed to determine the Maximum Non‐Toxic Concentration (MNTC) of DPSC‐CM. The MNTC is defined as the highest concentration of secretome that maintains cell viability above 70% compared to the untreated control group. An initial cytotoxicity screening was performed using an MTT (3‐(4, 5‐dimethylthiazol‐2‐yl) − 2, 5‐diphenyl tetrazolium bromide) assay (Sigma‐Aldrich Chemicals, Germany). Briefly, 3rd passage of PDLSCs were seeded in 96‐well plates at a density of 10^4^ cells/well in a growth medium (DMEM, 1% antibiotic, and 10% FBS). After 24 h, PDLSCs were treated with a range of DPSC‐CM concentrations (% V/V), spanning from 10% (V/V) up to 100% (V/V) (i.e., CM‐10, CM‐20, CM‐30, CM‐40, CM‐50, CM‐60, CM‐70, CM‐80, CM‐90, and CM‐100). Untreated PDLSCs cultured in the growth medium served as the control group.

This screening identified a threshold of 50% DPSC‐CM (V/V) as the MNTC and was therefore selected for subsequent functional experiment. A dose‐response curve was also plotted to enable the calculation of the inhibitory concentration by 50% (IC50), which is the concentration of the test compound that reduces the viability (absorbance) of the negative control by 50% (Shafiee et al. 2021).

### 2.9 MTT Protocol

At each designated time point (days 1, 3, and 7) for both screening and proliferation assays, the medium was removed, and cells were washed twice with PBS. Cells were then incubated with 100 μL MTT solution (5 mg/mL) for 2–4 h at 37°C. Following incubation, 150 μL of dimethyl sulfoxide (DMSO; Sigma‐Aldrich Chemicals, Germany) was added to dissolve the formed formazan crystals. The optical density (OD) was measured at 570 nm using a microplate reader (*n* = 3). Cell viability was calculated relative to the untreated control group using the following equation (Buranaamnuay 2021):

$$\text{Cell viability}\%=\;\;\frac{\text{Absorbance treated sample}}{\text{Absorbance control}}\;\;\times \;100.$$

### 2.10 Optimal Concentrations

The initial cytotoxicity screening indicated that secretome concentrations above 50% (V/V) were associated with a reduction in cell viability (< 70%). Therefore, based on the established MNTC and the need to investigate a wide functional range, three distinct concentrations were chosen to investigate the concentration‐dependent effects of DPSC‐CM on PDLSC proliferation, cytokine release, migration, ALP activity, and osteoblast differentiation as follows:

*High Dose (CM‐50)* 50% (V/V) DPSC‐CM, corresponding to approximately 100 μg/mL total protein.
*Medium Dose (CM‐30)* 30% (V/V) DPSC‐CM, corresponding to approximately 60 μg/mL total protein.
*Low Dose (CM‐10)* 10% (V/V) DPSC‐CM, corresponding to approximately 20 μg/mL total protein.

These concentrations are expressed using two metrics for clarity: the volumetric percentage of the crude secretome and its corresponding total protein concentration (as determined by BCA assay on the 100% (V/V) secretome stock). In addition, these concentrations were tested under two critical conditions for osteogenic induction: Growth Medium (GM) and Osteogenic Medium (OM), as detailed in Table 1.

**Table 1:** Group definition.

Groups ID	Cultured cell	Media base	Secretome concentration (V/V%)
GM^a^‐Control	PDLSCs^b^	GM^a^	—
GM^a^‐CM^c^ 50	PDLSCs^b^	GM^a^	50
GM^a^‐CM^c^ 30	PDLSCs^b^	GM^a^	30
GM^a^‐CM^c^ 10	PDLSCs^b^	GM^a^	10
OM^d^‐Control	PDLSCs^b^	OM^d^	—
OM^d^‐CM^c^ 50	PDLSCs^b^	OM^d^	50
OM^d^‐CM^c^ 30	PDLSCs^b^	OM^d^	30
OM^d^‐CM^c^ 10	PDLSCs^b^	OM^d^	10

^a^ GM: growth medium.

^b^ PDLSCs: periodontal ligament stem cells.

^c^ CM: Conditioned medium.

^d^ OM: Osteogenic medium.

### 2.11 Proliferation Assay

The effects of three optimal non‐toxic concentrations (V/V) of DPSC‐conditioned medium (CM) on periodontal ligament stem cell (PDLSC) proliferation were measured on Days 1, 3, and 7 using the MTT assay protocol (refer to the Cytotoxicity Screening section). The concentrations tested were 50% (CM‐50), 30% (CM‐30), and 10% (CM‐10).

### 2.12 Enzyme‐Linked Immunosorbent Assay (ELISA)

This part of the study was specifically conducted in growth medium (GM) to characterize the native paracrine factors released by DPSC‐CM that may induce migration and differentiation markers, independent of osteogenic supplements. PDLSCs at passage 3 (10^4^/well) were seeded in a 24‐well plate and cultured in GM (DMEM, 10% FBS, and 1% antibiotic) until they reached confluence. The medium was then replaced with fresh GM containing different concentrations (V/V) of DP‐CM (50%, 30%, and 10%), and the culture was continued for an additional 72 h. After this period, the cell culture supernatant was collected following centrifugation to remove cell debris.

The levels of Interleukin‐6 (IL‐6), transforming growth factor beta‐1 (TGF‐β1), and vascular endothelial growth factor (VEGF) in the culture supernatants were measured using Quantikine ELISA kits (R&D Systems) according to the manufacturer's instructions. Briefly, 200 µL of selected medium was added to 96‐well microplates coated with a monoclonal antibody specific to the factor of interest and incubated for 2 h. After washing with PBS, a horseradish peroxidase‐conjugated antibody specific to the cytokine was added to each well, followed by an additional 2 h incubation and washing. A substrate solution was then added and incubated for 30 min, after which the reaction was terminated by adding a stop solution. Cytokine levels were determined by measuring the optical density at 450 nm using a microplate spectrophotometer (Benchmark Plus; Bio‐Rad), and the results were expressed as pg/mL (*n* = 3).

### 2.13 Cell Migration Assay

A scratch assay was employed to evaluate the effects of different concentrations of DPSC‐CM on PDLSCs' migration. PDLSCs (20^4^ cells/well) were seeded in a 24‐well plate in GM (DMEM, 10% FBS, and 1% antibiotic) and incubated for 24 h at 37°C/5% CO_2_ to allow for cell adherence. A scratch was made in the cell monolayer with a sterile pipette tip in a straight line at the center of the plate. The cells were then washed with PBS to remove floating cells, and then treated with GM‐CM50, GM‐CM30, and GM‐CM10 for 48 h. PDLSCs cultured only in GM served as the control group. The migration of cells into the scratched area was observed using an inverted microscope, and images were taken at 24 and 48 h. This approach focused solely on GM to specifically isolate and quantify the DPSC‐CM's chemotactic potential on PDLSCs independent of external osteogenic cues. Based on Abcam's claims, the gap closure was measured and quantified using Image J software using the following equation:

$$\mathrm{Scratch}\;\mathrm{closure}\%=\frac{\mathrm{Scratch}\;\mathrm{width}\;\mathrm{time}\;0-\mathrm{scratch}\;\mathrm{width}\;\mathrm{time}\;1}{\mathrm{Scratch}\;\mathrm{width}\;\mathrm{time}\;0}\;\times 100$$

### 2.14 Osteoblastic Differentiation Induction

PDLSCs at passage 3 (5 × 10^3^ cells/well) were cultured in 24‐well plates in GM (DMEM, 10% FBS, and 1% antibiotic). After 24 h, the culture medium was replaced with OM (DMEM supplemented with 1% antibiotics, 10% FBS, 10 nmol/L dexamethasone, 50 µg/mL ascorbate phosphate, and 10 mmol/L b‐glycerophosphate). The cells were treated with different concentrations of DPSC‐CM for 14 days, and the culture medium was refreshed with the mentioned concentrations of DPSC‐CM twice a week.

### 2.15 Alkaline Phosphatase (ALP) Activity

The ALP activity assay was conducted using ALP activity kits (Pars Peyvand, Tehran, Iran). PDLSCs were lysed using RIPA buffer on days 7 and 14 after osteogenic induction. Briefly, the lysates were centrifuged (14,000 rpm/4°C/30 min), and 100 µL of the supernatant was mixed with 100 µl of pNPP (1 mg/mL) in 1 M diethanolamine buffer. This mixture was incubated for 15 min at 37°C. Subsequently, 12 µL of 3 N NaOH was added to stop the reaction. The supernatants were incubated with reaction buffer at 37°C for 15 min, and following the addition of a stop solution, the optical density was measured at 405 nm using a microplate reader. ALP activity of the samples was calculated according to the manufacturer's instructions (*n* = 3).

### 2.16 Real‐Time Polymerase Chain Reaction (RT‐PCR)

The mRNA expression of osteogenic/angiogenic markers, including ALP, bone morphogenic protein‐2 (BMP‐2), Runt‐related transcription factor 2 (RUNX‐2), osteopontin (OPN), and VEGF, was evaluated using RT‐PCR. Total RNA was extracted from cell cultures at 3, 7, and 14 days using Trizol reagent (Invitrogen, USA), followed by reverse transcription using a cDNA reverse transcription kit (Applied Biosystems, USA), in accordance with the manufacturer's instructions. Relative mRNA expression of the markers was evaluated using the ABI 7500 Real‐time PCR system (Applied Biosystems, CA, USA). The thermal cycling parameters were set as follows: Initial denaturation at 95°C for 10 min, followed by 40 cycles consisting of denaturation at 95°C for 15 s and annealing/extension at 60°C for 1 min.

The fold change in the expression of each marker was normalized against a housekeeping marker, glyceraldehyde‐3‐phosphate dehydrogenase (GAPDH), and calculated using the $2^{-∆∆C_{T}}$ method (Rao et al. 2013). RT‐PCR experiments were performed in triplicate. The primer sequences used are shown in Table 2.

**Table 2:** Nucleotide sequences of the primers used for RT‐PCR.

Biomarker	Primer	Sequences (5′ to 3′)
OPN^a^	Forward	TGGTCACTGATTTTCCCAC
	Reverse	TATCACCTCGGCCATCAT
ALP^b^	Forward	AGAAAGAGAAAGACCCCAAGTA
	Reverse	CCAGGAACATGATGACATTC
RUNX‐2^c^	Forward	GGGAACCCAGAAGGCACAG
	Reverse	AGGCGGGACACCTACTCTC
VEGF^d^	Forward	CCCATGGCAGAAGGAGGAG
	Reverse	GATGGCTTGAAGATGTACTCG
BMP‐2^e^	Forward	GAGGCAAAGAAAAGGAACGG
	Reverse	GCAGTCCGTCTAAGAAGCAC
GAPDH^f^	Forward	TCATCCATGACAACTTTGG
	Reverse	AGTCTTCTGGGTGGCAGT

^a^ OPN: osteopontin.

^b^ ALP: alkaline phosphatase activity.

^c^ RUNX‐2: Runt‐related transcription factor‐2.

^d^ VEGF: vascular endothelial growth factor.

^e^ BMP‐2: bone morphogenic protein‐2.

^f^ GAPDH: Glyceraldehyde‐3‐phosphate dehydrogenase.

### 2.17 Statistical Analysis

The statistical analysis was performed using SPSS software (version 18.0, SPSS Inc., Chicago, IL, USA). All experiments were conducted in triplicate, and data are displayed as mean values and standard deviations. The statistical differences between experimental groups at two‐time intervals were conducted using a Mann–Whitney *U* test. Multiple comparisons were performed using the Kruskal‐Wallis test, followed by the Dunn post hoc test. A *p*‐value < 0.05 was considered a significant level.

## 3 Results

### 3.1 PDLSCs Characterization

**Figure 1:**
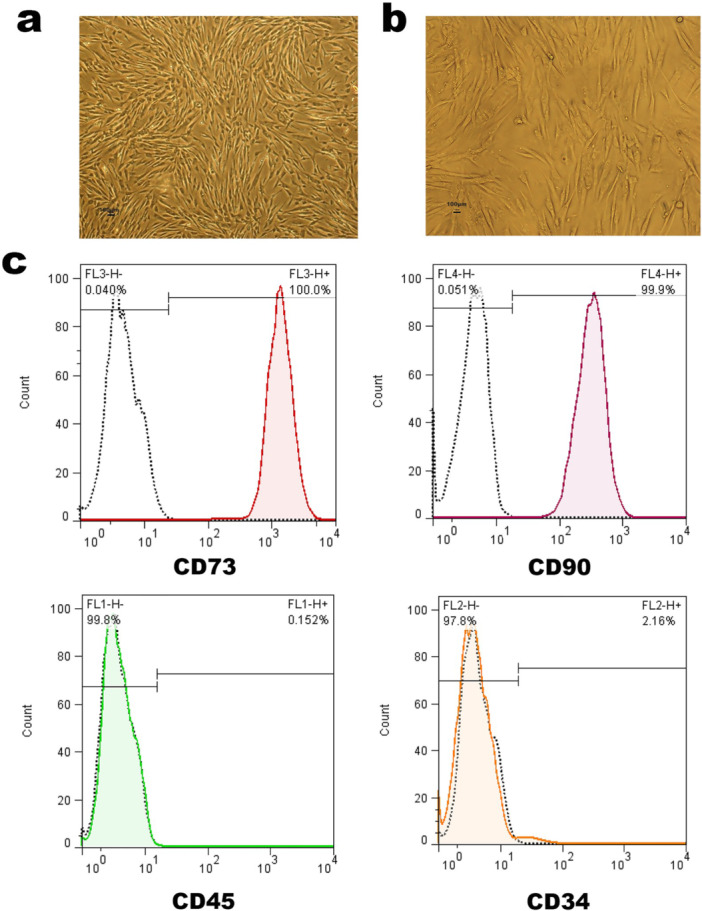
Characterization of isolated periodontal ligament stem cells (PDLSCs); (a) Morphology of the PDLSCs at passage 2 under inverted microscope (Magnification = 4X, scale bar= 100 µm); (b) Morphology of the PDLSCs at passage 2 under inverted microscope (Magnification = 10X, scale bar= 100 µm); (c) Flowcytometry histogram showing that isolated PDLSCs were positive for mesenchymal stem cells positive markers (CD‐73 and CD‐90) and negative for hematopoietic markers (CD‐34 and CD‐45).

The isolated cells displayed a spindle‐shaped fibroblast‐like morphology and had typical features of MSCs (Figure 1a,b). To confirm whether the isolated cells are indeed MSCs, the expression of mesenchymal and hematopoietic markers was evaluated using flow cytometry (Figure 1c). The results indicated a positive expression of mesenchymal markers and a negative expression of hematopoietic markers. The percentage of CD73‐positive and CD90‐positive cells were 97.8% and 99.8%, respectively. In contrast, the percentage of CD34‐negative and CD45‐negative cells were 0.04% and CD 0.051%, respectively.

### 3.2 Total Protein Concentrations

**Figure 2:**
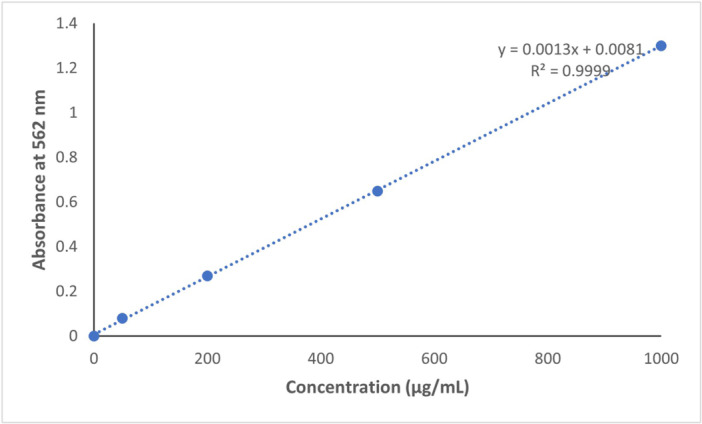
Absorbance curve of total protein concentration. The total protein concentration absorbance curve was calibrated using bovine serum albumin (BSA) standard.

The total protein concentration of the crude DPSC‐CM at 100% (V/V) was measured using the BCA assay and calibrated with the BSA standard (Figure 2). The total protein concentration was found to be 200 μg/mL.

### 3.3 Cytotoxic Screening/Cell Viability

**Figure 3:**
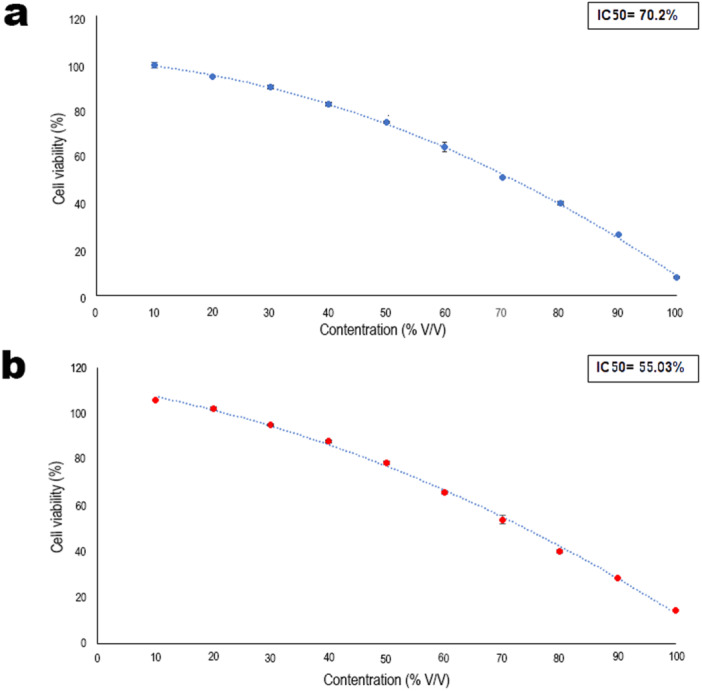
Cytotoxicity screening of different concentrations of dental pulp stem cells‐derived conditioned medium (DPSC‐CM) on periodontal ligament stem cells (PDLSCs) using MTT assay; (a) Day 1; (b) Day 3; PDLSCs were treated with 10 serial concentrations of DPSC‐CM, ranging from 10% (V/V) to 100% (V/V); data were analyzed using the non‐parametric Kruskal–Wallis test, followed by the Dunn post hoc test, and presented as the mean (SD) (*n* = 3).

The initial screening of different concentrations (V/V) of DPSC‐CM, ranging from 10% to 100%, was conducted to determine the MNTS. As shown in Figure 3, concentrations of DPSC‐CM equal to or above 50% (100 μg/mL) resulted in a significant reduction in cell viability (< 70%) at both Day 1 and Day 3, indicating cytotoxicity. IC50 was equal to 70.2% and 55.3% for days 1 and 3, respectively. Consequently, 50% (100 μg/mL) was established as the MNTS for further functional analyses, as cell viability remained above 70% at this concentration across all time points.

### 3.4 Optimal Concentrations

Based on the obtained results, three optimal non‐toxic concentrations (CM‐50, CM‐30, and CM‐10) were selected for downstream functional analyses. These analyses were performed under two distinct culture conditions to evaluate both the independent and synergistic effects of the secretome:

1. GM Conditions: Proliferation, Migration, and ELISA assays were performed solely under GM conditions.
2. Comparative Conditions (GM and OM): Osteoblast differentiation assays, including ALP activity and RT‐ PCR, were conducted under both GM and OM conditions.

### 3.5 Proliferation Assay

**Figure 4:**
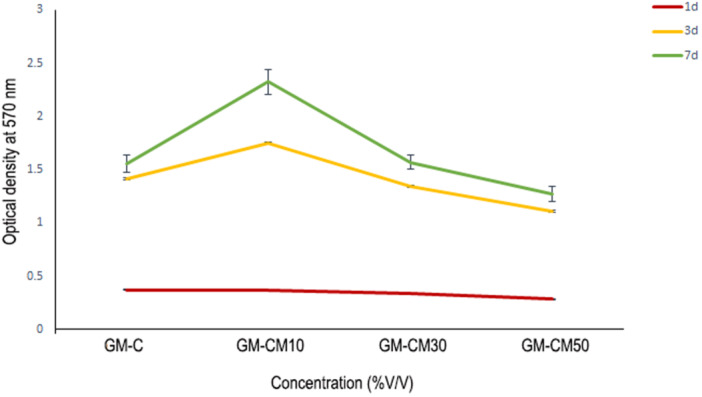
Proliferation of periodontal ligament stem cells treated with dental pulp stem cells‐derived conditioned medium at different concentrations (50% [GM‐CM50], 30% [GM‐CM30], and 10% [GM‐CM10]) up to 7 days using MTT assay. Data was analyzed using the non‐parametric Kruskal–Wallis test, followed by the Dunn post hoc test, and presented as the mean (SD) (*n* = 3) (*p* < 0.05).

The effect of the DPSC‐CM on PDLSC proliferation was assessed across the three optimal concentrations (10%, 30%, and 50%) over 7 days. Overall, cell metabolic activity, which indicates proliferation, significantly increased over time from Day 1 to Day 7 across all groups (*p* < 0.05), confirming the ongoing proliferative capacity of PDLSCs. However, the proliferative response to the DPSC‐CM was shown to be non‐linear and concentration‐specific (Figure 4). The lowest concentration (GM‐CM10; 20 µg/mL) demonstrated the most potent mitogenic effect, exhibiting a significantly higher proliferation rate than both the control group and the higher concentrations (GM‐CM30 and GM‐CM50) on all follow‐up days (days 1, 3, and 7; *p* < 0.05). Conversely, the higher concentrations (GM‐CM30 and GM‐CM50) displayed an inhibitory or cytostatic effect, showing significantly lower proliferation rates compared to the untreated control group at all time points (*p* < 0.05). No significant difference was observed in the proliferation rate between the GM‐CM30 and GM‐CM50 groups throughout the 7‐day culture period (*p* > 0.05).

### 3.6 ELISA Assay

**Figure 5:**
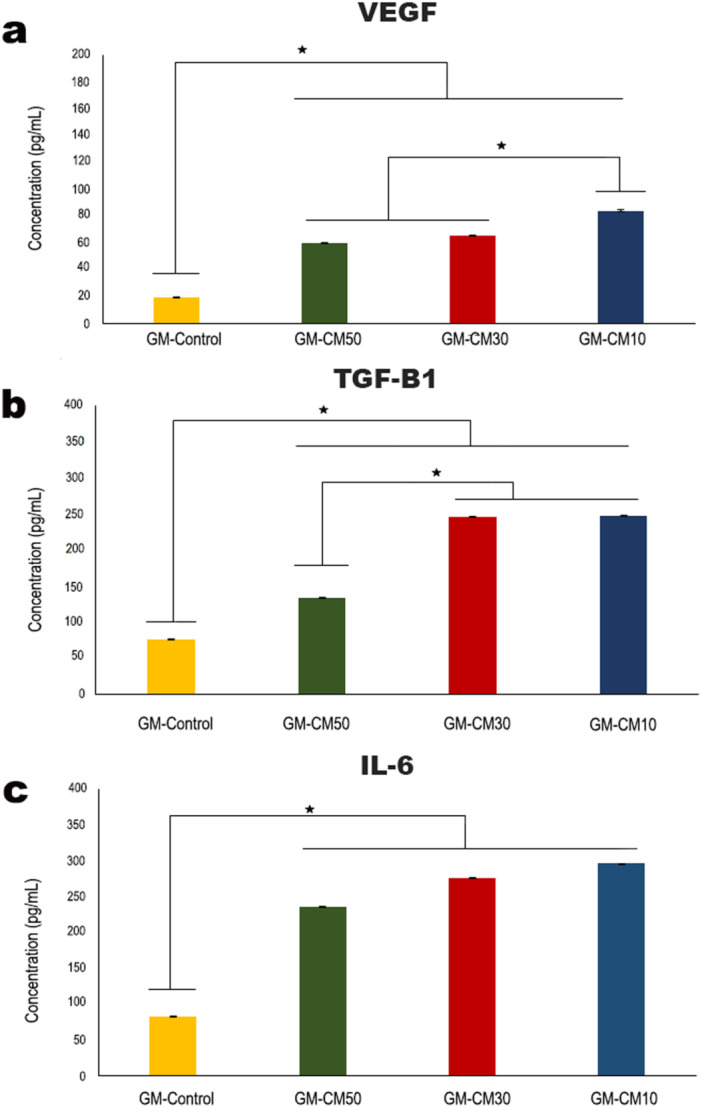
Cytokine profile in the culture media after 3 days culture of periodontal ligament stem cells with dental pulp stem cells‐derived conditioned medium at different concentrations (50% [GM‐CM50], 30% [GM‐CM30], and 10% [GM‐CM10]) using ELISA assay; (a) Vascular endothelial growth factor (VEGF); (b) Transforming growth factor‐Betha 1 (TGF‐β1); (c) Interleukin‐6 (IL‐6). The expression level is expressed as pg/mL; data were analyzed using a non‐parametric Kruskal–Wallis test, followed by the Dunn post hoc test and presented as the mean (SD) (*n* = 3); black asterisks represent the significant difference between the groups (*p* < 0.05).

The concentrations of specific cytokines (IL‐6, TGF‐β1, and VEGF) released into the GM supernatant by PDLSCs were quantified using ELISA (Figure 5). In comparison to the control group, the concentrations of all three quantified cytokines (IL‐6, TGF‐β, and VEGF) were significantly higher in all secretome‐treated groups (*p* < 0.05).

Among the three factors, IL‐6 exhibited the highest overall concentration, followed by TGF‐β1 and VEGF (*p* < 0.05). The intergroup analysis revealed distinct concentration‐dependent patterns:

IL‐6: No significant differences in IL‐6 concentration were observed between the CM‐50, CM‐30, and CM‐10 groups (*p* > 0.05), suggesting that saturation of IL‐6 secretion occurs even at the lowest dose.
TGF‐ β: Expression levels of TGF‐β1 in the lower concentrations (CM‐30 and CM‐10) were significantly higher compared to the highest dose (CM‐50) (*p* < 0.05). No significant differences were found between the CM‐30 and CM‐10 groups (*p* > 0.05).
VEGF: Similar to TGF‐β1, the lowest dose, CM‐10, resulted in the highest concentration of VEGF. The VEGF level in the CM‐10 group was significantly greater than in the CM‐30 and CM‐50 groups (*p* < 0.05).

### 3.7 Cell Migration Assay

**Figure 6:**
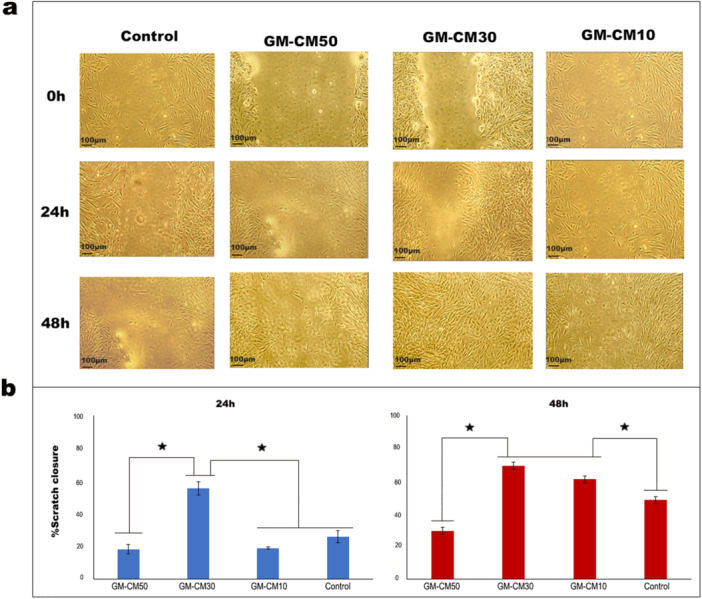
Migration of periodontal ligament stem cells treated with dental pulp stem cells‐derived conditioned medium at different concentrations (50% [GM‐CM50], 30% [GM‐CM30], and 10% [GM‐CM10]) up to 48 h using the scratch assay. (a) Scratch assays of PDLSCs over 48 h. Images were taken at 0, 24, and 48 h after scratching using inverted microscope (Magnification = 4X, scale bar = 100 µm); (b) Quantitative data representing % scratch closure at 24 and 48 h relative to 0 h; data were analyzed using non‐parametric Kruskal–Wallis test, followed by the Dunn post hoc test and presented as the mean (SD) (*n* = 3); black asterisks represent the significant difference between the groups (*p* < 0.05).

To assess the migration potential of DPSC‐CM, a scratch assay was performed at 24 and 48 h, correlating with the baseline (0 h) (Figure 6).

The migration rate of PDLSCs increased significantly at 48 h compared to 24 h (*p* < 0.05). In addition, the DPSC‐CM exhibited a concentration‐specific effect on PDLSCs migration, indicating the existence of an optimal chemotactic concentration. When comparing different concentrations, GM‐CM30 showed significantly higher migration than GM‐CM10 at 24 h; however, this difference was non‐significant at 48 h. GM‐CM30 also demonstrated significantly higher migration than GM‐CM50 at both 24 and 48 h, while GM‐CM10 exhibited significantly higher migration than GM‐CM50 only at 48 h. Compared to the control, a significant difference was observed between GM‐CM30 and the control group at 24 and 48 h, and between GM‐CM10 and the control group only at 48 h.

### 3.8 ALP Activity Assay

**Figure 7:**
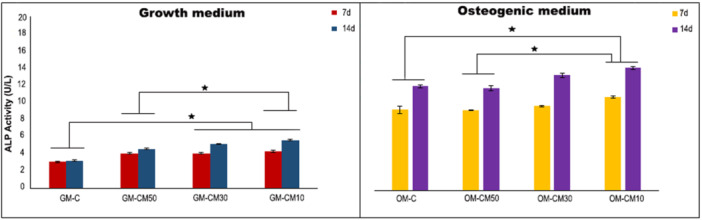
Alkaline phosphatase (ALP) activity of periodontal ligament stem cells treated with different concentrations of dental pulp stem cells‐derived conditioned medium under osteogenic medium (OM) (50% [OM‐CM50], 30% [OM‐CM30], and 10% [OM‐CM10]) and growth medium (GM) (50% [GM‐CM50], 30% [GM‐CM30], and 10% [GM‐CM10]) up to 14 days. The expression level is the ALP enzyme activity (U/L); data were analyzed using the non‐parametric Kruskal–Wallis test, followed by the Dunn post hoc test, and presented as the mean (SD) (*n* = 3); black asterisks represent the significant difference between the groups (*p* < 0.05).

ALP activity was measured as an indicator of early‐mid osteogenic commitment in PDLSCs, following treatment with three optimal non‐toxic concentrations in both GM (i.e., GM‐CM50, GM‐CM30, and GM‐CM10) and OM conditions (i.e., OM‐CM50, OM‐CM30, and OM‐CM10) at Days 7 and 14 (Figure 7). The results revealed a clear time‐dependent increase in osteogenic activity: ALP activity was significantly higher at Day 14 compared to Day 7 across all experimental groups (*p* < 0.05).

### 3.9 ALP Activity Under OM Conditions

Under OM conditions, only OM‐CM10 significantly enhanced ALP activity compared to the OM Control group at both Day 7 (*p* = 0.002) and Day 14 (*p* = 0.002). This finding confirmed a synergistic effect of the secretome when combined with the osteogenic cocktail.

Analysis of concentration effects revealed a pattern consistent with the non‐linear response observed in the proliferation assay. The lowest dose, OM − CM10, demonstrated the maximal osteogenic effect, showing a significant increase in ALP activity compared to the highest concentrations (OM − CM50) at both Day 7 and Day 14 (*p* < 0.05). At both time points, no significant differences were detected between OM‐CM30 with either OM‐CM10 (*p* > 0.05) or OM‐CM50 (*p* > 0.05).

### 3.10 ALP Activity Under GM Conditions

Under GM conditions, ALP activity in the GM‐CM10 group was significantly higher than in the GM control (*p* < 0.05), indicating that DPSC‐CM has an inherent capacity to induce osteogenesis even without traditional osteogenic supplements. A non‐linear concentration‐specific response was evident; the GM‐CM10 group exhibited maximum ALP activity at both Day 7 (*p* = 0.002) and Day 14 (*p* = 0.003) compared to the higher concentrations (GM‐CM30 and GM‐CM50). There was no significant difference between GM‐CM50 and GM‐CM30 at either time point (*p* > 0.05), suggesting that these higher doses provide comparable, sub‐optimal osteogenic induction.

### 3.11 Comparison of OM Versus GM Conditions

When comparing the two culture environments, ALP activity in the OM condition was significantly greater than in the GM condition for all tested concentrations at both time points (*p* < 0.05). This confirms that while the secretome is an effective osteogenic inducer on its own, its full potential is realized when combined with a conventional osteogenic medium.

### 3.12 Gene Expression Analysis (RT‐PCR)

The expression levels of early osteogenic markers (RUNX‐2, BMP‐2), mid‐late osteogenic markers (ALP, OPN), and the angiogenic marker (VEGF) were evaluated in PDLSCs, following treatment with three optimal non‐toxic concentrations under both GM (GM‐CM50, GM‐CM30, and GM‐CM10) and OM (OM‐CM50, OM‐CM30, and OM‐CM10) conditions. The expression levels of the mid‐late markers were evaluated on days 7 and 14, while the expression of early osteogenic and angiogenic markers was evaluated on days 3, 7, and 14.

The results revealed a clear time‐dependent pattern; the overall expression levels of ALP and OPN increased by Day 14 compared to Day 7 (*p* < 0.05). Moreover, the expression levels of BMP‐2, VEGF, and RUNX‐2 increased by Day 3 (*p* < 0.05), peaked by Day 7 (*p* < 0.05), and then decreased by Day 14 (*p* < 0.05), indicating a temporal regulation of early commitment genes.

### 3.13 Early Osteogenic Markers

**Figure 8:**
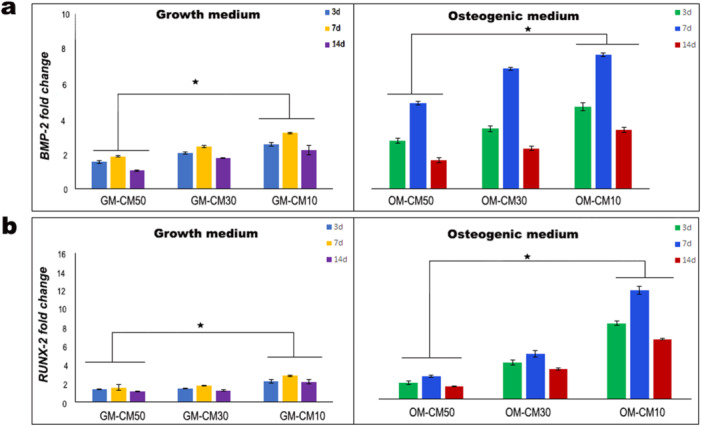
Relative expression of early osteogenesis markers of periodontal ligament stem cells treated with different concentrations of dental pulp stem cells‐derived conditioned medium under osteogenic medium (OM) (50% [OM‐CM50], 30% [OM‐CM30], and 10% [OM‐CM10]) and growth medium (GM) (50% [GM‐CM50], 30% [GM‐CM30], and 10% [GM‐CM10]) up to 14 days; (a) Runt‐related transcription factor 2 (RUNX‐2); (b) Bone morphogenic protein‐2 (BMP‐2); the expression level is the fold change relative to the control group; data were analyzed using a non‐parametric Kruskal‐Wallis test, followed by the Dunn post hoc test and presented as the mean (SD) (*n* = 3); black asterisks represent the significant difference between the groups (*p* < 0.05).

The expression of early osteogenic markers was evaluated on Days 3, 7, and 14 (Figure 8). Under OM conditions, the gene expression showed a concentration‐specific effect, with lower doses generally leading to better induction; the expression of BMP‐2 and RUNX‐2 was significantly upregulated in the OM‐CM10 group compared to OM‐CM50 (*p* < 0.05). No significant difference was detected between the OM‐CM30 and OM‐CM10 groups (*p* > 0.05), nor between the OM‐CM30 and OM‐CM50 groups (*p* > 0.05). A concentration‐specific effect on gene expression was also observed under GM conditions, with the pattern similar to OM conditions; BMP‐2 and RUNX‐2 were significantly upregulated in GM‐CM10 compared to GM‐CM50 (*p* < 0.05). No significant difference was detected between GM‐CM30 and either GM‐CM10 (*p* > 0.05) or GM‐CM50 groups (*p* > 0.05), suggesting that this intermediate concentration yielded a non‐statistically distinguishable effect from both the optimal and sub‐optimal doses.

### 3.14 Mid‐Late Osteogenic Markers

**Figure 9:**
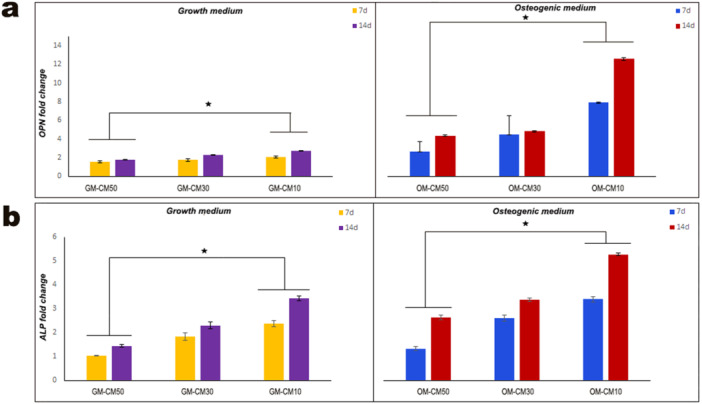
Relative expression of mid‐late osteogenesis markers of periodontal ligament stem cells treated with different concentrations of dental pulp stem cells‐derived conditioned medium under osteogenic medium (OM) (50% [OM‐CM50], 30% [OM‐CM30], and 10% [OM‐CM10]) and growth medium (GM) (50% [GM‐CM50], 30% [GM‐CM30], and 10% [GM‐CM10]) up to 14 days; (a) Alkaline phosphatase (ALP), (b) Osteopontin (OPN); the expression level is the fold change relative to the control group; data was analyzed using a non‐parametric Kruskal‐Wallis test, followed by the Dunn post hoc test and presented as the mean (SD) (*n* = 3); black asterisks represent the significant difference between the groups (*p* < 0.05).

The expression of mid‐late osteogenic markers was evaluated on Days 7 and 14 (Figure 9). Under OM conditions, the lowest dose (OM‐CM10) showed significantly higher expression levels than the higher doses (OM‐CM30 and OM‐CM50) (*p* < 0.05). However, no significant difference was observed between OM‐CM30 and either OM‐CM10 or OM‐CM50 (*p* > 0.05). Under GM conditions, GM‐CM10 showed significantly higher expression levels than GM‐CM50 (*p* < 0.05). However, no significant difference was detected between GM‐CM30 and either GM‐CM10 or GM‐CM50 (*p* > 0.05).

### 3.15 Angiogenic Markers

**Figure 10:**
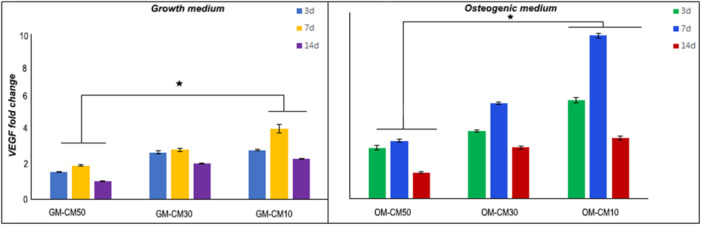
Relative expression of angiogenic markers (vascular endothelial growth factor [VEGF]) of periodontal ligament stem cells treatment with different concentrations of dental pulp stem cells‐derived conditioned medium under osteogenic medium (OM) (50% [OM‐CM50], 30% [OM‐CM30], and 10% [OM‐CM10]) and growth medium (GM) (50% [GM‐CM50], 30% [GM‐CM30], and 10% [GM‐CM10]) up to 14 days; the expression level is the fold change relative to the control group; data was analyzed using non‐parametric Kruskall–wallis test, followed by the Dunn post hoc test and presented as the mean (SD) (*n* = 3); black asterisks represent the significant difference between the groups (*p* < 0.05).

The expression of angiogenic markers was evaluated on days 3, 7, and 14 (Figure 10). Under OM conditions, VEGF expression was highest in the OM‐CM10 group, showing significant upregulation compared to OM‐CM50 (*p* < 0.05). Furthermore, no significant difference was noted between OM‐CM30 and either OM‐CM10 (*p* > 0.05) or OM‐CM50 groups (*p* > 0.05). Under GM conditions, VEGF expression was highest in the GM‐CM10 group, with significant upregulation compared to the GM‐CM50 (*p* < 0.05), and no significant difference was noted between GM‐CM30 and either GM‐CM10 (*p* < 0.05) or GM‐CM50 (*p* > 0.05).

### 3.16 Comparison of OM Versus GM Conditions

When comparing the two culture environments, expression of the osteogenic/angiogenic markers in the OM condition was significantly higher than in the GM condition for all tested concentrations and at all time points (*p* < 0.05). This confirms that while the secretome is an effective osteo‐inducer on its own, its full potential is realized when combined with a conventional osteogenic medium.

## 4 Discussion

In the current study, for the first time, we aimed to evaluate the osteoinductive effect of DPSC‐CM on PDLSCs, focusing on its efficacy in a dose‐dependent manner. We assessed the differentiation capacity of DPSC‐CM under two distinct conditions: GM and OM. The GM condition, which lacks specific pro‐osteogenic supplements, such as dexamethasone, β‐Glycerophosphate, and ascorbic acid, was used to test the inherent inductive potential of the secretome. Conversely, the OM condition allowed us to assess the synergistic effects of DPSC‐CM combined with the strong chemical stimuli present in a traditional differentiation cocktail. Our findings from both the ALP activity assay and the RT‐PCR analysis consistently showed that DPSC‐CM significantly enhanced the expression of osteogenic markers (including ALP, RUNX2, and OPN) in the GM condition compared to the GM Control. However, the highest levels of osteogenic induction were achieved in the OM condition. This indicates that while the paracrine factors in the secretome are effective osteoinducers on their own, their maximum therapeutic effect—and likely their superior translational potential—occurs when they act together with a supportive osteogenic microenvironment.

We also explored the promising application of DPSC‐CM therapy and found that DPSC‐CM positively influenced the proliferation, migration, and osteogenic differentiation of PDLSCs. Furthermore, it showed potential to promote the expression of angiogenic markers in PDLSCs in a concentration‐specific manner, which deviates from a simple linear dose‐response. The effects of the secretome on cellular activities can significantly vary depending on the concentration of trophic factors and inhibitory by‐products. Thus, it was important to define the optimal concentration of the secretome to achieve the necessary balance for biological function. In our MTT assay, we evaluated a range of concentrations (20–200 μg/mL) and confirmed that concentrations above 100 μg/mL significantly reduced cell viability. In line with our findings, Paschalidis et al. found that DPSC‐CM at 100% dilution decreased cell viability compared to a 50% dilution under similar culture conditions (Paschalidis et al. 2014). According to our viability assay, we selected three concentrations for further evaluations: (CM‐50 [100 µg/mL], CM‐30 [60 µg/mL], and CM‐ 10 [20 µg/mL]). Notably, across most functional assays—such as Proliferation, ALP activity, and expression of osteogenic markers—the lowest concentration, CM − 10 (20 μg/mL), demonstrated the most beneficial effects, supporting a hormesis‐like effect rather than a simple dose‐dependent trend.

PDLSCs treated with DPSC‐CM showed a significant increase in their proliferation rate over 7 days, with the effects being concentration‐specific. Notably, significant results were observed only in the group treated with CM‐10 (20 µg/mL). This enhanced proliferation may result from specific concentrations of mitogens and antiapoptotic factors, including VEGF and TGF‐β1, which may have synergistic effects within the DPSC‐CM. Considering the clinical application of this finding, it can be assumed that CM transplantation into periodontal defects on a biocompatible scaffold (Nagata et al. 2017) can enhance the proliferation rate of PDLSCs, the main stem cell source for periodontal regeneration, which can lead to shorter probable healing time and near‐optimum tissue maturation. In our ELISA assay, we detected the presence of VEGF and TGF‐β1 in the 3‐day culture; both of which are known to promote cell proliferation, filopodial extensions, and extracellular matrix degradation. In a study by Gharaei et al. DPSC‐CM similarly promoted the proliferation of human umbilical vein endothelial cells at concentrations of 25% and 50% (V/V). Conversely, another study reported no significant effects on the proliferation of DPSCs when treated with 25% and 50% BMSCs‐CM (Nagata et al. 2017). Our results also found that DPSC‐CM at concentrations of 30% and 50% (V/V) did not significantly affect the proliferation rate of PDLSCs. These contrasting results could be ascribed to variation in the concentrations of trophic factors produced by various cell types, which may also depend on the specific cell culture condition used.

Our migration assay revealed a unique concentration‐dependent effect. On Day 1, only the intermediate concentration, CM − 30 (60 μg/mL), significantly enhanced the migratory capacity of PDLSCs compared to all other groups. However, by Day 2, both CM‐10 and CM‐30 showed similar migratory effects. Migration is a complex process requiring a precise balance of chemotactic factors, which might differ from those needed for cell proliferation. It seems that by Day 2, both CM‐10 and CM − 30 provide an optimal balance of chemotactic factors, such as VEGF, which act as stimulants for migration.

In the current study, we selected ALP activity and several osteogenic markers, including RUNX‐2, BMP‐2, Col‐1, and ALP, to evaluate the osteoblast differentiation. ALP is an important marker for the early to mid stages of osteogenic differentiation and plays a crucial role in the maturation of hard tissues (Ansari et al. 2022). Runx2 and BMP‐2 are key transcription factors involved in osteogenesis; they serve as early indicators and are essential for the production of bone matrix proteins and osteoblast differentiation (Komori 2003). Additionally, OPN is a marker that becomes important in the later stages of mineralization (Sodek et al. 1995).

Our ALP activity assay showed that treatment with CM‐10 (20 μg/mL) significantly enhanced ALP activity at both 7 and 14 days, indicating improved mineralization. This finding once again indicated not only the dose‐dependent effect of different concentrations of DPSC‐CM, but also the positive outcome of the synergistic effect of CM‐10 in OM. This observation is consistent with Kumar's study, which reported increased levels of BMP7 in DPSCs‐CM that facilitate mineralization (Kumar et al. 2018). In addition, our RT‐PCR results indicated that DPSC‐CM at a concentration of 10% (V/V) increased the expression levels of osteogenic markers, including Runx2, OPN, BMP‐2, and ALP. BMP2, a member of the TGF‐β superfamily, is an osteogenic marker; the binding of BMP type I and II receptors to ligands on cell surfaces may lead to the osteoblastic differentiation of MSCs (Zhang et al. 2019; Phimphilai et al. 2006). Similarly, another study has also shown that the BMP2/Runx2 axis regulates osteoblastic activity, with BMP signaling enhancing Runx2 activity and vice versa (Marconi et al. 2020).

The consistent findings across our functional assays—including proliferation, ELISA, ALP activity, and gene expression (where the lower dose, CM − 10, outperformed the higher dose, CM − 50)—strongly suggest a non‐linear dose‐response (hormesis‐like effect). This pattern implies that DPSC‐CM at its highest concentration may contain factors that shift the cellular response from promotion to inhibition. We hypothesize two potential mechanisms for the reduced efficacy observed in the CM‐50 group: First, the higher concentration may contain increased levels of metabolic by‐products or inhibitory cytokines released by the DPSCs, which can suppress PDLSC differentiation pathways or increase cellular stress. Second, the excessive concentration of stimulating paracrine factors in CM‐50 may lead to the downregulation or desensitization of key cellular receptors (such as TGF‐β or VEGF receptors) on the PDLSCs. This downregulation would prevent the cells from responding effectively to the signal, resulting in a sub‐optimal functional outcome despite the presence of high levels of trophic factors. This critical balance underscores the necessity of defining a precise therapeutic window for acellular secretome therapies.

Furthermore, we found that treating PDLSCs with DPSC‐CM could increase the expression of cytokines, such as IL‐6, VEGF, and TGF‐β1, in the 3‐day cell culture. This may synergistically impact the migration, angiogenesis, and osteogenic differentiation of PDLSCs. The effects of these three cytokines on the biological activities are believed to be highly complex.

Pro‐inflammatory cytokines are known to have immunomodulatory effects during periodontal wound healing, with IL‐6 recognized as a key pro‐inflammatory cytokine. In addition, IL‐6 has been shown to positively affect osteoblastic differentiation (Iwasaki et al. 2008). Some researchers have suggested that IL‐6 plays a functional role in bone formation, particularly in studies using STAT‐1/3 and MAPK transgenic mice models (Sims et al. 2004). Besides, PDLSCs predominantly contain collagen I, which has a high turnover rate (Yamada et al. 2001). This suggests that IL‐6/sIL‐6R could enhance the osteoblastic differentiation of PDLSCs in periodontal lesions. In our study, all three concentrations of CM led to elevated levels of IL‐6 after 3 days. Since IL‐6 has shown both pro‐inflammatory and anti‐inflammatory functions, it is known to have dual effects on bone homeostasis (Wang et al. 2023). This feature should be valued in explaining the healing of periodontal bone defects, as it accelerates new bone formation through the induction of BMP9 expression. In a study by Al‐Sharabi et al. an increase in IL‐6 levels was observed in the supernatant from DPSC‐CM, which is consistent with our findings (Al‐Sharabi et al. 2017). Conversely, Nagata et al. examined the effect of PDLSC‐CM on periodontal defects in rats and reported a non‐significant reduction in IL‐6 levels during the wound healing process. Although these findings do not align with ours, the non‐significant reduction in IL‐6 expression may be attributed to variation in the formulation of CM and the timing of evaluation; it may be more effective to conduct this assessment at earlier stages of healing (Nagata et al. 2017).

TGF‐β1 has been shown to regulate the proliferation, migration, and osteogenic differentiation of MSCs (Ramoshebi et al. 2002; Mathieu et al. 2013). Our ELISA assay showed that the expression of TGF‐β1 was significantly greater in the CM‐10 and CM‐30 groups than in the CM‐50 and control groups. Given that TGF‐β1 has been shown to exert chemotactic effects on osteoblasts and to stimulate osteogenic differentiation, it appears that lower concentrations of DPSCs‐CM may enhance bone regeneration.

It has been reported that DPSC‐CM can enhance angiogenesis by releasing various pro‐angiogenic factors. VEGF is a master regulator of angiogenesis, promoting the survival and differentiation of endothelial cells, which in turn supports osteogenesis (Chakka et al. 2021; Artese et al. 2010). As shown in our ELISA assay, treatment with DPSC‐CM significantly increased VEGF expression levels within the first 3 days in the CM‐10 group (60 µg/mL) compared to higher concentrations. This finding was further confirmed by RT‐PCR, which showed that extracellular VEGF secretion followed a similar pattern in mRNA gene expression analysis. A key aspect of angiogenesis is cellular migration along a chemotactic gradient through the extracellular matrix in the remodeled perivascular space. Data from the migration assay showed that the CM‐30 group (60 µg/mL) exhibited the highest migration capacity on Day 1 compared to the other groups. However, the migration capacity of the CM‐10 and CM‐30 was the same by Day 2. We speculate that this may be attributed to a dysregulation of functional protein activity. Additionally, the synergic effects of the released cytokines in the culture medium may play a role; for instance, TGF‐β can upregulate VEGF synthesis in endothelial cells (Krishnan et al. 2015).

However, there are limitations to our experiment that should be noted. First, we identified several key cytokines by ELISA; however, future studies should employ comprehensive proteomic analysis (e.g., mass spectrometry) to identify the full secretome profile, including any potential inhibitory byproducts. By this approach, we may explain the reduced efficacy observed in the CM‐50 group and the increased efficacy detected in the CM‐10 group. Second, it is essential to design an animal study to assess the efficiency and safety of different DPSC‐CM concentrations in a periodontal defect. Third, developing a secretome‐loaded delivery system could be beneficial for future clinical applications.

In conclusion, the current research demonstrates that DPSC‐CM may serve as a promising osteoinductive agent for enhancing bone repair in periodontal regeneration. The soluble factors released from DPSCs can promote proliferation, migration, and osteogenesis. Importantly, these effects were concentration‐specific and non‐linear. The optimal dose for proliferation and the expression of osteogenic/angiogenic markers is found at CM‐10, while CM‐10 and CM‐30 are optimal for migration after 2 days. This strongly suggests that the high proliferative and paracrine activity of CM‐10 directly translates into superior osteogenic induction, while the higher doses may be inhibitory or sub‐optimal due to saturation or the activation of counter‐regulatory pathways.

## Author Contributions

Substantial contribution to the conception and design of the study: Mahdi Kadkhodazadeh, Aida Kheiri. Substantial contribution to the acquisition, interpretation, and analysis of data: Reza Amid, Aida Kheiri, Sayna Shamszadeh, and Forough Shams. Drafting the article or revising it critically for the important intellectual content: Aida Kheiri, Mahdi Kadkhodazadeh, Reza Amid, Sayna Shamszadeh. Final approval of the version to be published: Mahdi Kadkhodazadeh, Reza Amid, Aida Kheiri, Sayna Shamszadeh, Forough Shams.

## Funding

The authors have nothing to report.

## Ethics Statement

The protocol for isolating human periodontal ligament stem cells (hPDLSCs) was approved by the University of Murcia Ethics Committee (IRB number: 3686/2021). The current research was approved by the Ethics Committee of the Dental School at Shahid Beheshti University of Medical Sciences under approval number IR.SBMU.DRC.REC.1402.079 (Approval date: 2023‐08‐01). Since the present study did not experiment on human or animal subjects, there were no ethical issues regarding human and animal rights.

## Consent

Each participant provided written consent prior to their involvement in the research.

## Conflicts of Interest

The authors declare no conflicts of interest.

## Supporting information

Supporting File 1.

Supporting File 2

## Data Availability

The data that support the findings of this study are available from the corresponding author upon reasonable request.
