# Supplementary material for: Osteogenic Differentiation Induced by Dental Pulp Stem Cells Secretome: A Dose‐Dependent Comparative Study
Source: Clin Exp Dent Res. 2026 Jun 23;12(3):e70395. doi: 10.1002/cre2.70395 (PMC13290014; doi:10.1002/cre2.70395)
Supplement: Supplementary file 1 — Supporting File 1. [file CRE2-12-e70395-s002.docx]

| **Secretome concentration (V/V%)** | **Protein concentration**  **(**μg/mL**)** |
| --- | --- |
| - | - |
| 50 | 100 |
| 30 | 60 |
| 10 | 20 |
| - | - |
| 50 | 100 |
| 30 | 60 |
| 10 | 20 |

**Table 3 (supplementary). Protein concentrations in three different secretome concentrations**
